# The assessment of Intolerance of uncertainty in youth: An examination of the Intolerance of Uncertainty Scale-Revised in Italian nonclinical boys and girls

**DOI:** 10.1007/s10802-022-00944-y

**Published:** 2022-07-02

**Authors:** Gioia Bottesi, Sara Iannattone, Eleonora Carraro, Marco Lauriola

**Affiliations:** 1 Department of General Psychology, University of Padova, via Venezia 8, 35131 Padova, Italy; 2 Department of Psychology of Developmental and Socialisation Processes, Sapienza University of Rome, via dei Marsi 78, 00185, Rome, Italy

**Keywords:** Intolerance of uncertainty, Adolescence, Assessment, Factor structure, Psychometrics

## Abstract

Intolerance of Uncertainty (IU) is a transdiagnostic factor involved in several psychological disorders. Adolescence is characterized by elevated uncertainty and psychopathological vulnerability, but insufficient attention has been paid to IU at this age. This study aimed to investigate the factor structure and psychometric properties of the Intolerance of Uncertainty Scale-Revised (IUS-R) in Italian preadolescents and adolescents. 862 Italian students (57.3% girls) aged 11–17 (*M* = 14.8 ± 1.91) completed the IUS-R and measures of internalizing and externalizing symptoms, and psychological well-being. To test the factor structure of the IUS-R, one-factor, two-factor, and bifactor models were compared; measurement invariance, reliability, and validity were also addressed. Results showed that the bifactor model outperformed alternative factor models, and a general factor was needed to model the IUS-R. Bifactor model indices supported using the total score to assess IU reliably. Configural and metric invariance by age and sex were fully supported, while the IUS-R achieved partial scalar invariance. Significant correlations emerged for the IUS-R total score with psychopathological constructs, while no relationships with psychological well-being were found. Compared to adult normative data, higher total IUS-R scores were detected, suggesting that IU may be a phase-specific characteristic of adolescence. Our findings support using the IUS-R to measure IU across the lifespan. The recommended use of the total score and its associations with psychopathological dimensions highlight the transdiagnostic nature of IU in adolescence. Therefore, including IU when implementing interventions to prevent maladaptive outcomes in teenagers would be beneficial.

**Supplementary Information:**

The online version contains supplementary material available at 10.1007/s10802-022-00944-y.

Intolerance of Uncertainty (IU) is a trait-like disposition reflecting “the tendency to be bothered or upset by the (as yet) unknown elements of a situation, whether the possible outcome is negative or not” (Freeston et al., 2020, p. 6). People high in IU struggle to tolerate and modulate the negative emotions elicited by uncertainty (i.e., uncertainty distress) and usually experience negative emotional, behavioral, and cognitive responses when dealing with uncertain situations (Freeston et al., 2020).

IU was originally conceptualized as a cognitive vulnerability factor for worry, the core feature of Generalized Anxiety Disorder (GAD; Dugas et al., 1998; Freeston et al., 1994). It is now widely recognized that, while IU remains central in GAD, it has broad applications across a wide range of psychopathologies, such as anxiety disorders, obsessive-compulsive disorder, depression, post-traumatic stress disorder, eating disorders, substance use disorders, and personality disorders (see Bottesi et al., 2018; 2021; Gentes & Ruscio, 2011; Shihata et al., 2016). Furthermore, recent evidence supports the role of IU as a transdiagnostic and trans-therapy change process (McEvoy et al., 2019a; McEvoy & Erceg-Hurn, 2016), and treatment protocols focusing on IU when targeting different psychological disorders have proved effective (e.g., Shapiro et al., 2020).

Nevertheless, the literature about the transdiagnostic nature of IU is mainly based on studies conducted in adult samples. Insufficient attention has been paid to the role of IU in youth (Osmanağaoğlu et al., 2018; Shihata et al., 2016), although most psychological disorders have their onset in adolescence (e.g., Polanczyk et al., 2015; Sanchez et al., 2017). This gap in the literature is questionable given that adolescents face novel, changeable, and uncontrollable events, scattered by lots of uncertainties in different areas (e.g., sexuality, education, identity, interpersonal relationships, relative independence from parents) (Casey et al., 2010). Moreover, adolescence is a stage of brain development characterized by the maturation of the neural pathways associated with inhibitory control (i.e., regulation of emotion and behavior) and cognitive beliefs about the future, uncertainty, and threat (Read et al., 2013). Therefore, exploring IU in adolescence is crucial since it may help increase knowledge about the developmental trajectories of psychopathologies that have their onset in this period of life, such as anxiety disorders, substance use disorders, and eating disorders (De Girolamo et al., 2012).

## The Assessment of IU in Adolescence

Since 1994 a vast body of literature has focused on IU measurement, and most research has been conducted on undergraduate and adult samples (Bottesi et al., 2020). The Intolerance of Uncertainty Scale-12 (IUS-12; Carleton et al., 2007) is the shortened version of the original 27-item Intolerance of Uncertainty Scale (IUS-27; Freeston et al., 1994), and it is the most widely used self-report measure assessing IU in adulthood (Bottesi et al., 2019b; McEvoy et al., 2019a). Initial research addressing the factor structure of the IUS-12 suggested that it encompasses two separate dimensions: Prospective IU, referring to the predisposition for active information seeking to reduce uncertainty, and Inhibitory IU, expressing avoidance-oriented reactions to uncertainty (Birrell et al., 2011; Carleton et al., 2007; McEvoy & Mahoney, 2011). However, the most recent literature agrees that a general factor should be preferred to the two canonical factors to improve the adequacy of factor analysis models and support the use of the total score instead of two overly intercorrelated subscale scores (Bottesi et al., 2019b; Hale et al., 2016; Huntley et al., 2020; Lauriola et al., 2016; Shihata et al., 2018; Yao et al., 2021; Wilson et al., 2020).

The factor structure of the IUS-12 in a nonclinical sample of adolescents was first explored by Boelen et al. (2010). Data from participants aged 14–18 were analyzed, and a two-factor structure emerged from Principal Component Analysis. However, two of the seven items deemed to reflect the Prospective IU factor (i.e., #1 and #2) loaded higher on the Inhibitory IU factor, suggesting the need for an additional general factor, as found for adult samples. A Dutch study also investigated the factor structure, reliability, and validity of the IUS-12 in a sample of adolescents (age range: 13–17) (Dekkers et al., 2017). The two-factor model fitted the data better than the one-factor model, and the Prospective and Inhibitory IU factors were found to have a good internal consistency reliability. Furthermore, the two factors were partially invariant across sex and were positively associated with trait anxiety and negatively related to risk-taking in cognitive tasks.

### The Intolerance of Uncertainty Scale for Children (IUSC)

The first attempt to develop a scale suitable for administration in children and adolescents is the Intolerance of Uncertainty Scale for Children (IUSC; Comer et al., 2009), a downward extension of the IUS-27 (Freeston et al., 1994) with simplified instructions and items to enhance child compatibility. The IUSC was psychometrically evaluated in a mixed sample of youth (nonclinical and with an anxiety disorder) aged 7–17. The total score showed an excellent internal consistency in nonclinical and clinical samples, large positive correlations with anxiety and worry, and a moderate correlation with reassurance-seeking behavior (Comer et al., 2009). More recently, Cornacchio et al. (2018) examined the factor structure of the IUSC and its abridged 12-item version (IUSC-12) in a youth sample aged 9–18 with and without anxiety disorders. Although the bifactor model fitted the data significantly better than the two-factor model, the study questioned the use of the total score in clinical research, given the marginally better performance of the Prospective and Inhibitory IU subscales in predicting anxiety and depression scores in concurrent validity analyses (Cornacchio et al., 2018). Recently, Osmanağaoğlu et al. (2021) tested both the IUSC versions in a nonclinical sample of children and preadolescents (age range: 7.58–11.81). The IUSC-12 was overall more appropriate than the IUSC for this age group. Unlike previous research, the study concluded that a one-dimensional model with only 11 items would be more parsimonious than a corresponding bifactor model. However, the bifactor model would not be inferior to the one-factor model in terms of absolute fit and parsimony without the atheoretical removal of item #10 (i.e., “One should always think ahead to avoid surprises”).

### The Intolerance of Uncertainty Scale-Revised (IUS-R)

Almost contemporary to the IUSC is the Intolerance of Uncertainty Scale-Revised (IUS-R; Walker et al., 2010). The IUS-R is a refinement of the IUS-12 (Carleton et al., 2007), with a simplified language to be easily read by an average 11-year-old student (Walker et al., 2010). The IUS-R was designed to assess IU across the lifespan: using the same scale with people of different ages would make it easier to understand how IU evolves over the life course and aid in integrating findings from different studies (McEvoy et al., 2019b). The English version was used in healthy young people, children, and adolescents with diagnoses of Autism or Asperger’s syndrome (e.g., Boulter et al., 2014; Joyce et al., 2017; Wright et al., 2016). However, the factorial structure was not addressed in youth samples, except for preliminary findings from eight British and Spanish nonclinical groups (i.e., 6–8 years; 9–11 years; 12–14 years; undergraduate students) (Bottesi & Freeston, 2012). In these samples, the two-factor model sufficiently fitted the data; however, the bifactor model was not tested, as was usually the case in less recent studies of the IUS scales. Moreover, Bottesi and Freeston (2012) did not address the convergent/discriminant validity of the IUS-R. In a subsequent study, the two-factor model fitted the data better than the one-factor model in Italian undergraduates, and the IUS-R demonstrated good internal consistency values (Bottesi et al., 2015). Although the study supported the sex invariance of the IUS-R, the bifactor model was again not investigated. More recently, however, Bottesi et al. (2019b) explored the factor structure of the IUS-R in an Italian community sample, showing that a bifactor model outperformed the two-factor model in terms of model fit and achieved the standard of measurement invariance across sex, age, and over time.

## The Current Study

Adolescence is a period characterized by high vulnerability to psychopathology, and IU is a transdiagnostic factor involved in several mental disorders emerging in this life stage. The existing scales for evaluating IU in this population are promising, but there are still some issues. For instance, the IUSC studies provided inconsistent findings regarding the factorial structure and used relatively small mixed samples of nonclinical and clinical children and adolescents (e.g., Comer et al., 2009; Cornacchio et al., 2018; Osmanağaoğlu et al., 2021). Instead, the IUS-R was found to have a robust factor structure in undergraduate and adult samples only, despite its intent to assess IU across the lifespan (Bottesi et al., 2019b). The current study was designed to fill these gaps, exploring the factor structure and psychometric properties of the IUS-R in a large sample of Italian preadolescents and adolescents (age range: 11–17).

Since the IUS-R is a refined and linguistically simplified version of the IUS-12, our research hypotheses were based on the extant literature on the original version of the scale in young-adult and community samples. First, we expected to confirm the bifactor model and support using the total score for assessment purposes. In addition to the bifactor model, we considered the one- and two-factor models for comparison, as these models were considered in the evaluation of IU in youth. Second, we sought to investigate the measurement invariance across sex and age groups (i.e., preadolescence, early adolescence, and middle adolescence), an issue rarely explored in the literature. Although measurement invariance is frequently assumed, it is also often overlooked, even though it is a prerequisite for comparing group means. If measurement invariance is attained, the IUS-R items have the same meaning for preadolescents, early and middle adolescents, or girls and boys; consequently, the construct can be measured with the same measurement unit, baseline level, and precision across age and sex groups. Lastly, we expected the IUS-R to show moderate-strong correlations with internalizing symptoms and weak negative or no significant correlations with measures of general psychological well-being (i.e., the Psychological Well-Being Scale and the Positivity Scale; see the “Measures section” for details). Specifically, the Psychological Well-Being Scale is a measure derived from the eudaimonic theory (Ryff & Keyes, 1995), which defines well-being as experiencing a sense of realization of personal potential. This definition is different from hedonic wellbeing, which is the presence of pleasurable, positively-valenced feelings, such as life satisfaction or happiness. Instead, the Positivity Scale assesses the disposition to evaluate life with a positive attitude. Therefore, we considered these two measures adequate to examine the divergent validity of the IUS-R.

## Method

### Participants

The sample was made up of 862 Italian youth (494 girls, 57.3%) aged 11 to 17 (*M* = 14.8 ± 1.91). Of these, 24.7% were recruited from lower secondary schools and 75.3% from upper secondary schools located in midsize cities in northern Italy. Specifically, with regard to the former, 30.5% attended the Italian first class (6th grade), 37.1% the second class (7th grade), and 32.4% the third class (8th grade). Among high school students, 14.6% attended the first class (9th grade), 22.7% the second class (10th grade), 31.4% the third class (11th grade), and 31.2% the fourth and fifth classes (12th grade). Participants were asked whether they had ever experienced psychological problems for which they requested professional help. Among those who responded (*n* = 808), 11% reported current or past psychological difficulties, such as anxiety, depression, eating disorders, learning difficulties (e.g., dyslexia), and family or school problems. The mean total score obtained by participants on the IUS-R was 31.8 (*SD* = 9.25), while the mean scores on the other administered questionnaires are presented in the Supplementary Information (Table S1).

## Procedure

The present study is part of a broader longitudinal research project about the role of IU as a transdiagnostic risk factor for the onset of psychological disorders in adolescence. The study was approved by the Ethics Committee for Psychological Research of the University of Padova and was conducted in accordance with the Declaration of Helsinki.

The data considered in this work were collected from November 2018 to November 2019 at lower and upper secondary schools in northern Italy. The project was first presented to school directors to obtain their approval. Subsequently, all parents (or legal guardians) signed a written informed consent form, and students gave their assent. No incentives or rewards were offered for participating. There were no exclusion criteria. An ad hoc online survey – composed of a socio-demographic form and a battery of self-report tools (see list below) – was administered to the sample during regular school hours. Students filled in the survey in their school’s computer room, and it took approximately 45/50 minutes in each class. Confidentiality of data treatment was guaranteed by using an identification code created by each student at the beginning of the survey.

## Measures

The online survey included the following questionnaires:

- The Intolerance of Uncertainty Scale-Revised (IUS-R; Walker et al., 2010) comprises 12 items. Participants are asked to rate each item on a five-point Likert scale (1 = *not at all agree*, 5 = *completely agree*). The Italian version showed an excellent internal consistency (Cronbach’s α *=* 0.90, Bentler’s ω *=* 0.90, McDonald’s ω_h_ = 0.90) and a good one-month test-retest reliability (*r* = 0.74) in undergraduate and adult samples (Bottesi et al., 2019b).
- The *Youth Self Report 11–18* (YSR; Achenbach & Rescorla, 2001) is a self-report questionnaire widely used to assess emotional and behavioral problems in young people aged 11 to 18. It comprises 112 items rated on a three-point Likert scale (0 = *not true*; 1 = *sometimes true*; 2 = *very true*). Problem behaviors are identified through eight syndrome scales: anxiety/depression, withdrawal/depression, somatization, social problems, thought-related problems, attention problems, aggressive behavior, and rule-breaking behavior. These scales can be grouped into problem areas like internalizing (i.e., anxiety, depression, withdrawal, and somatization) and externalizing (i.e., aggressive, and rule-breaking behavior). In the present study, Cronbach’s alpha coefficients for each scale ranged from 0.69 to 0.90, consistently with the original Italian version of the tool (Cronbach’s α ranging from 0.83 to 0.91) (Frigerio et al., 2004).
- The *Self-Administered Psychiatric Scales for Children and Adolescents – Anxiety Scale* (SAFA-A; Cianchetti & Fancello, 2001) is an Italian self-report tool that measures anxiety symptoms in children and adolescents aged 8 to 18. The versions for preadolescents and adolescents consist of 50 items grouped into four subscales: generalized anxiety (composed of the tension/uneasiness and preoccupation-apprehension for the future subscales), social anxiety, separation/loss anxiety, and school-related anxiety. Three possible responses are provided for each item: *true*, *partly true*, and *false*. The original version of the tool showed good test-retest stability (*r* > 0.75) and internal consistency (Cronbach’s α > 0.85) (Cianchetti & Fancello, 2001); an adequate reliability of each subscale emerged in the present study, too (Cronbach’s α between 0.78 and 0.89).
- The *Positivity Scale* (P Scale; Caprara et al., 2012) is a self-report questionnaire assessing positivity, a general disposition to evaluate life and experiences with a positive attitude. It comprises 8 items - rated on a five-point Likert scale (1 = *strongly disagree*, 5 = *strongly agree*). Although the P Scale evaluates different aspects of positivity (i.e., self-esteem, life satisfaction, positive expectation about the future, and confidence in receiving support from others), its unidimensionality has been supported in adolescent samples (Tian et al., 2018; Zuffianò et al., 2019). The psychometric properties of the Italian questionnaire are adequate, with good internal consistency (Cronbach’s α = 0.75) and test-retest reliability (*r* = 0.76) (Caprara et al., 2012). The tool achieved satisfactory reliability in the present study, with Cronbach’s α = 0.85.
- The *Psychological Well-Being Scales – 18 item version* (PWB; Ryff & Keyes, 1995; Italian version by Ruini et al., 2003) is a self-rating questionnaire composed of 18 items assessing six dimensions of psychological well-being: self-acceptance, autonomy, environmental mastery, personal growth, purpose in life, and positive relations. Participants indicate their responses on a six-point Likert scale (1 = *strongly disagree*, 6 = *strongly agree*). The factorial structure of the PWB has been also supported with Italian adolescents, and the tool showed adequate psychometric properties (Cronbach’s α ranging from 0.60 to 0.70) (Sirigatti et al., 2009; Viola et al., 2016). Given the purpose of the present study, we only considered the PWB total score, which showed a good internal consistency (Cronbach’s α = 0.86).

## Statistical Analyses

### Factor models

We tested a one-factor model, a two-factor model with correlated factors, and a bifactor model with a general factor and two specific factors. The one-factor model assumed that each item reflected only a common latent IU factor, whereas item uniqueness reflected an unspecified mixture of random and systematic error variance. Such an assumption is often unrealistic because many systematic factors impact item responses in self-report scales. The two-factor model assumed that the IU domain could be divided into Prospective IU (items 1–7) and Inhibitory IU (items 8–12). Item responses are thought to reflect relatively independent common factors. As above, uniqueness reflected a mixture of random and systematic error variance. Most IUS-R research supported a bifactor model with three orthogonal factors: Inhibitory IU, Prospective IU, and General IU. The former two factors parse random error variance from the variance common to specific groups of items. The general IU factor reflects the common variance to all items and represents the trait underlying core IU beliefs above and beyond Prospective and Inhibitory IU.

### Confirmatory factor analysis (CFA)

The “lavaan” and “semTools” packages for R were employed to estimate parameters and test hypotheses about CFA models (Jorgensen et al., 2018; Rosseel, 2012). All factor models were fitted using Maximum Likelihood Robust (MLR) estimators. The assessment of the model fit was based on the MLRχ2 and other recommended indices (i.e., CFI, TLI, RMSEA, and SRMR). CFI and TLI values > 0.95 indicate a good fit, while values > 0.90 are acceptable. An RMSEA of 0.06 or less is a good fit, while values < 0.08 are acceptable. The SRMR < 0.08 supports a good fit between the model and the data (Schermelleh-Engel et al., 2003). To find a baseline model for measurement invariance analyses, we compared the fit of the one-factor, two-factor, and bifactor models. For this purpose, a chi-square difference test can be performed if the models are nested. This condition is met for the one-factor and two-factor models, both nested in the bifactor model (Reise, 2012), and the two-factor model, which is nested in the one-factor model.

### Bifactor Model Indices

The usefulness of a bifactor model for understanding the properties of a psychometric scale can be assessed using specific indices (see Rodriguez et al., 2016). The Percentage of Uncontaminated Correlations (PUC) measures the proportion of item correlations that reflects only the general factor variance (Rodriguez et al., 2016). The Explained Common Variance (ECV) assesses the proportion of variance in the items explained by the general factor relative to the total amount of shared variance explained. According to Rodriguez et al. (2016), when both PUC and ECV are greater than 0.70, the scale under investigation can be regarded as unidimensional, and the factor loadings for the one-factor model and the general factor in a bifactor model will not be substantially different. Other convenient indices are ω and ω_h_. The former measures the overall proportion of reliable variance in the IUS-R total score accounted for by the general and group factors. The latter only reflects the proportion of reliable variance in the IUS-R total score due to the general factor. Minor differences between reliability coefficients (ω and ω_h_) support using the IUS-R total score to measure IU in youth.

### Measurement invariance

We tested the measurement invariance of the IUS-R by stages of adolescence and sex groups. First, we compared preadolescent (11–13 years), early adolescent (14 and 15 years), and middle adolescent (16 and 17 years) groups. Girls and boys were compared in a subsequent set of analyses. First, we tested whether the equality in factor structure is supported in other groups (Configural Invariance). Provided a good fit for the configural model, we tested whether the factor loadings were equal across groups (Metric Invariance). Then, we constrained both the factor loadings and item intercepts to be equal across groups (Scalar Invariance). Each level of invariance can be compared to the next using the chi-square difference test. Invariance is supported if the test turns out not statistically significant. However, because trivial differences might yield statistically significant differences, Chen (2007) recommended considering a difference in CFI ≤ 0.010, paired with differences in RMSEA and SRMR ≤ 0.015 as criteria to support the substantial equivalence of model fit.

### Construct validity

Pearson’s *r* correlations were computed with the SAFA-A and YSR scales to test the convergent validity of the IUS-R. With regard to divergent validity, Pearson’s *r* correlations were calculated between the IUS-R and the PWB and P Scale. Following Cohen’s (1988) criteria, *r* = 0.10 indicates a small effect, *r* = 0.30 a moderate effect, and *r* = 0.50 a large effect. For ANOVA, the effect size was assessed using Cohen’s *f*, with 0.10, 0.25, and 0.40 representing small, medium, and large effect sizes, respectively (Cohen, 1988). The IUS-R score was also the dependent variable in three regression analyses with general psychological well-being, SAFA-A, and YSR scales as predictors. The R^2^ was the effect size measure used in these analyses, with 0.01, 0.09, and 0.25 representing small, medium, and large effects, respectively.

## Results

### Factor structure of the IUS-R

Table 1 reports the fit indices for different CFA models of the IUS-R. All fit indices were good. As expected, the one-factor model was a poor fit. The two-factor model significantly improved over the one-factor model, producing acceptable-to-good fit indices. However, the Prospective and Inhibitory IU factors were highly correlated (*Φ* = 0.83), casting doubts on the substantial independence of the two latent variables (Van Mierlo et al., 2009). The bifactor model significantly improved the model’s fit compared to the two-factor model. A non-significant RMSEA indicated a close fit for this model, and the other fit indices were excellent.

**Table 1:** Fit indices for the Confirmatory Factor Analysis models of the IUS-R

	Bifactor Model	Two-Factor Model	One-Factor Model
χ^2^*(df)*	111.89^***^ (42)	217.04^***^ (53)	315.87^***^ (54)
CFI	0.97	0.94	0.90
TLI	0.96	0.92	0.87
RMSEA	0.04	0.06	0.08
95%CI	0.04 − 0.05	0.05 − 0.06	0.07 − 0.08
*p*-close	0.846	0.015	0.000
SRMR	0.03	0.05	0.05
∆χ^2^(*df*)	---	85.66^***^ (11)	86.80^***^ (1)
∆CFI	---	− 0.04	− 0.04
∆RMSEA	---	0.02	0.02
∆SRMR	---	0.02	0.01
*Note.* * *p* < 0.05, ** *p* < 0.01, *** *p* < 0.001. χ2 = scaled chi square; df = degrees of freedom; ∆χ2 = scaled chi square difference test; CFI = Comparative Fit Index; TLI = Tucker-Lewis Index; RMSEA = Root Mean Square Error of Approximation; 95%CI = 95% confidence interval for Root Mean Square Error of Approximation; *p*-close = RMSEA close fit test; SRMR = Standardized Root Mean Square Residual; χ2 = chi square; ∆χ2(*df*) = chi square difference test; ∆CFI = change in Comparative Fit Index; ∆RMSEA = change in Root Mean Square Error of Approximation; ∆SRMR = change in Standardized Root Mean Square Residual.			

The standardized factor loading matrix is reported in Table 2. All items significantly loaded on the General factor, which explained 32% of the total variance. Except for item #3, all factor loadings for the General factor were in the 0.42 – 0.73 range. The Prospective and Inhibitory factors accounted for smaller fractions of the total variance (3% and 7%, respectively). Only three items out of seven significantly loaded the Prospective IU factor (λ_range_ = 0.19 –0.54). Five items out of five loaded significantly on the Inhibitory IU factor (λ_range_ = 0.13 –0.54). The ECV equal to 0.75 achieved Rodriguez et al. (2016) criterion (i.e., ECV > 0.70) for preferring the total score to subscale scores; however, the PUC = 0.53 recommended caution. In this case, an inspection of model-based reliability coefficients is needed to recommend using the total score instead of separate subscale scores. The ω and ω_h_ for the general factor were close (0.88 and 0.80, respectively). For Prospective IU, the ω_h_ was remarkably lower than the corresponding ω (0.77 and 0.04, respectively). Likewise, the ω_h_ was lower than ω for Inhibitory IU (0.83 and 0.24, respectively). These findings showed that the Prospective and Inhibitory IU factors did not contribute substantially to the total score’s reliability. Conversely, the reliability of the subscales was mostly due to the General factor variance, especially for Prospective IU. Collectively, these findings show the IUS-R total score is viable and preferable to subscale scores.

**Table 2:** Standardized factor loadings and uniqueness for the Bifactor Model of the IUS-R

Item	General		Prospective		Inhibitory		*u* ^2^
1 – When things happen suddenly, I get very upset.	0.73	^***^	− 0.23		---		0.42
2 – It bothers me when there are things I don’t know.	0.51	^***^	0.01		---		0.75
3 – People should always think about what will happen next. This will stop bad things from happening.	0.33	^***^	0.26	^***^	---		0.83
4 – Even if you plan things really well, one little thing can ruin it.	0.47	^***^	0.09		---		0.77
5 – I always want to know what will happen to me in the future.	0.42	^***^	0.19	^***^	---		0.79
6 – I can’t stand it when things happen suddenly.	0.72	^***^	0.06		---		0.48
7 – I should always be prepared before things happen.	0.60	^***^	0.54	^***^	---		0.35
8 – Feeling unsure stops me from doing most things.	0.59	^***^	---		0.47	^***^	0.43
9 – When I’m not sure what to do I freeze.	0.52	^***^	---		0.54	^***^	0.44
10 – When I don’t know what will happen, I can’t do things very well.	0.64	^***^	---		0.28	^***^	0.51
11 – The smallest concern can stop me from doing things.	0.61	^***^	---		0.45	^***^	0.42
12 – I must get away from all things I am unsure of.	0.55	^***^	---		0.13	^*^	0.68
*Note.* * *p* < 0.05, ** *p* < 0.01, *** *p* < 0.001.							

The ANOVA of the IUS-R total score showed statistically significant differences by sex (*F*_1,856_ = 7.78; *p* < 0.01; Cohen’s *f* = 0.09) and age group (*F*_2,856_ = 5.10; *p* < 0.01; Cohen’s *f* = 0.09), whereas the interaction was not significant. Girls’ scores (*M* = 32.56; *SD* = 9.32) were significantly higher than boys’ scores (*M* = 30.66; *SD* = 9.07). Gabriel’s post hoc test (suitable for unequal sample sizes) showed that the preadolescent group (*M* = 33.26; *SD* = 9.30) obtained higher IUS-R scores than early (*M* = 30.75; *SD* = 8.85) and middle (*M* = 31.61; *SD* = 9.41) adolescents, although mean differences were only marginally significant in the second comparison (*p*_Gabriel_ = 0.009 and 0.097, respectively). No differences were found comparing the two adolescent groups (*p*_Gabriel_ = 0.565). The following measurement invariance section will address whether the observed differences reflect true differences in the latent variable or item-specific variance components unrelated to IU.

## Measurement invariance of the IUS-R

The bifactor model was the baseline model in multigroup analyses (MGCFA) aimed at testing the measurement invariance of the IUS-R. Fit indices and hypothesis testing are reported in Table 3.

**Table 3:** Tests of Measurement Invariance by age and sex groups

	MGCFA by age			MGCFA by sex		
	Configural Invariance	Metric Invariance	Scalar Invariance^1^	Configural Invariance	Metric Invariance	Scalar Invariance^2^
χ^2^(*df*)	187.14^***^(126)	249.01^***^(174)	297.2^***^(195)	137.32^***^(84)	146.26^***^(108)	196.86^***^(116)
CFI	0.98	0.97	0.97	0.98	0.99	0.98
TLI	0.96	0.97	0.97	0.97	0.98	0.97
RMSEA	0.04	0.04	0.04	0.04	0.03	0.04
95%CI	0.03 − 0.06	0.03 − 0.05	0.03 − 0.05	0.03 − 0.05	0.02 − 0.04	0.03 − 0.05
*p*-close	0.896	0.969	0.925	0.966	0.999	0.992
SRMR	0.03	0.05	0.06	0.03	0.04	0.05
∆χ^2^(*df*)	---	62.12^†^(48)	47.82^***^(21)		14.08(24)	38.00^***^(8)
∆CFI	---	− 0.01	− 0.01		0.00	− 0.01
∆RMSEA	---	− 0.00	0.00		− 0.01	0.01
∆SRMR	---	0.02	0.01		0.01	0.01
*Note.* † p < 0.10, * p < 0.05, ** p < 0.01, *** p < 0.001. χ2 = scaled chi square; df = degrees of freedom; ∆χ2 = scaled chi square difference test; CFI = Comparative Fit Index; TLI = Tucker-Lewis Index; RMSEA = Root Mean Square Error of Approximation; 95%CI = 95% confidence interval for Root Mean Square Error of Approximation; *p*-close = RMSEA close fit test; SRMR = Standardized Root Mean Square Residual; χ2 = chi square; ∆χ2(*df*) = chi square difference test; ∆CFI = change in Comparative Fit Index; ∆RMSEA = change in Root Mean Square Error of Approximation; ∆SRMR = change in Standardized Root Mean Square Residual. ^1^ Item intercepts were freed for items #2, #5 and #12 in the early adolescent group ^2^ Item intercepts were freed for items #1, #7, #8, and #12						

The configural and metric invariance models were excellent in MGCFA analyses by sex and age. The chi-square difference test between models was only marginally significant (*p* < 0.10) in the analysis by age, and the differences in other fit indices were below the thresholds for concluding no substantial loss of fit (Chen, 2007). In the analysis by sex, the chi-square difference test was not statistically significant. These results supported the metric invariance hypothesis. In contrast, the scalar invariance hypothesis was rejected because models that imposed the equality of item intercepts significantly lost fit compared to metric invariance models. Latent mean comparisons become equivocal when scalar invariance fails. A difference in the latent means could be due to a difference in the item intercepts rather than to a difference in the IU construct.

The inspection of the model modification indices revealed that the source of the misfit in the analysis by age was due to the non-invariance of a few item intercepts (i.e., #2, #5, and #12) between the preadolescent group and the two adolescent groups. Likewise, noninvariant item intercepts (i.e., #1, #7, #8, and #12) produced misfit in the analysis by sex. Allowing those intercepts to be freely estimated for the preadolescent and female groups supported the partial scalar invariance by age and sex (i.e., *∆*CFI ≤ 0.010, *∆*RMSEA and *∆*SRMR ≤ 0.015).

Partial scalar invariance is deemed insufficient for interpreting latent mean differences without ambiguity (e.g., De Beuckelaer & Swinnen, 2018; Kuha & Moustaki, 2015). Nevertheless, because the intercepts of early and middle adolescents were invariant, we investigated how these age groups differed in the General IU factor to aid the interpretation of ANOVA results previously reported. A derivative of the partial scalar invariance model was tested, freeing the latent means of the preadolescent and early adolescent groups. Thus, the standardized parameter for each group is expressed as a Standardized Mean Difference from the middle adolescent group that serves as reference group. The derivative model fitted the data significantly better than the partial invariance model (*∆*χ^*2*^ *=* 10.31; *df* = 2; *p* < 0.001), but the latent mean of the early adolescent group did not differ from the reference group (*SMD* = − 0.14; *z* = -1.59; *p* = 0.112).

## Construct validity of the IUS-R

The correlation matrix between the IUS-R and the other administered questionnaires is displayed in the Supplementary Information (Table S2). As regards convergent validity, moderate-to-high positive correlations emerged with all the SAFA-A scales (Pearson’s *r* ranging from 0.31 to 0.54) and with the YSR anxiety/depression, withdrawal/depression, social problems, thought-related problems, and internalizing problems scales (Pearson’s *r* ranging from 0.30 to 0.51). Low and positive correlations were detected with the YSR somatic complaints, aggressive behavior, attention problems, and externalizing problems scales (Pearson’s *r* ranging from 0.10 to 0.28), while the only non-significant correlation was observed with the rule-breaking behavior scale. Then, pertaining to divergent validity, no significant correlation emerged considering the PWB total score, while a small negative correlation was found with the P Scale. Regression analyses (Supplementary Table S3) showed that the SAFA-A and YSR scales accounted for 34% and 28% of the variance in IUS-R scores, respectively. The SAFA-A generalized anxiety and YSR anxiety/depression scores were the single best predictors of IU scores. The variables used to assess the divergent validity accounted for a minimal amount of the IUS-R variance (3%) and only the P Scale was significant. Thus, regression analyses confirmed the convergent and divergent validity of the IUS-R.

## Discussion

The transdiagnostic nature of IU and its role in the onset of psychological disorders are well-established in adulthood (e.g., McEvoy et al., 2019a). In contrast, the IU construct has received insufficient attention during adolescence, although this period of life is fraught with uncertainty, and vulnerability to psychopathology is high (e.g., Bottesi, in press); unsolved issues about IU measurement also plague IU research in teenagers (Cornacchio et al., 2018; Shihata et al., 2016). Bearing all this in mind, the present study aimed to investigate the factorial structure and psychometric properties of the gold standard tool for measuring IU – namely, the IUS-R – in a sample of Italian youth. The study’s main findings confirmed the hypotheses and added to our understanding of the characteristics and evaluation of IU in adolescence as follows.

The correlated two-factor model showed acceptable fit indices. Nevertheless, the Prospective and Inhibitory IU factors were highly correlated, thus suggesting empirical overlap. The bifactor model significantly improved the fit, demonstrating that the IUS-R items are influenced by a broad general factor and two narrow group factors reflecting Prospective and Inhibitory IU. This broad factor accounted for 75% of the common variance, an amount consistent with previous studies conducted with adult samples, in which it ranged from 75 to 87% (Bottesi et al., 2019b; Hale et al., 2016; Huntley et al., 2020; Lauriola et al., 2016; Shihata et al., 2018; Wilson et al., 2020; Yao et al., 2021).

Recent studies have questioned the bifactor model’s validity in youth, recommending instead using the unidimensional model after the atheoretical removal of one item (Osmanağaoğlu et al., 2021), or the two-factor model, which performed better in concurrent validity analyses (Cornacchio et al., 2018). Nonetheless, since these studies used a different IUS scale (i.e., the IUSC), it is unclear whether negative findings regarding the bifactor model are due to that specific scale or whether the general factor is problematic to be found and measured at this age. Our results are consistent with the first interpretation, given that the bifactor model outperformed the competing models of the IUS-R without removing any items, and partly agreed with Osmanağaoğlu et al. (2021) regarding the use of the total score as a reliable assessment of the IU construct in adolescence.

Item loadings on the General factor are in line with findings obtained in nonclinical adult samples, being moderately high and statistically significant (Bottesi et al., 2019b; Hale et al., 2016; Huntley et al., 2020; Lauriola et al., 2016; Shihata et al., 2018). A notable exception was the factor loading of item #3, which was barely acceptable in magnitude, consistently with previous studies in Italian adult samples (Bottesi et al., 2019b; Lauriola et al., 2016). Our result may thereby constitute additional evidence of the occurrence of cross-cultural differences in interpreting uncertainty. Such an issue is still unexplored by the extant literature. However, it has been speculated that differences in how the cultures engage with uncertainty at the level of IU core beliefs may occur (Bottesi et al., 2019b; Lauriola et al., 2018) and that culturally different attitudes to metacognition may intervene in the interpretation of uncertainty (Bottesi et al., 2016). Studies in the field of economics and business sciences listed Italy among the “strong uncertainty avoidance cultures”, thus suggesting that Italians are more likely to react negatively to unfamiliar situations than individuals with a different cultural background (e.g., Stremersch & Tellis, 2004). Therefore, a cultural variation in response to items assessing coping with uncertainty through deliberative processes may reflect the role played by cultural values in defining the way individuals interpret and express their emotions.

Nonetheless, neurocognitive development in adolescence should be considered, too. Indeed, item #3 explicitly refers to the need to plan to avoid future unpleasant events (i.e., “People should always think about what will happen next. This will stop bad things from happening.”), so it seems to imply deliberative processes to cope with uncertainty. Frontal and prefrontal brain areas - responsible for planning complex cognitive behaviors, rational decision-making, and moderation of social conduct - are still underdeveloped during adolescence (Spear, 2000), thus reducing the teenagers’ ability to plan rationally. Therefore, adolescents’ peculiar neuropsychological and cognitive functioning may explain why they did not fully endorse item #3. This is further supported by the results of Osmanağaoğlu et al. (2021), who found that item #10 of the IUSC-12, concerning the need to look ahead to prevent surprises, was judged difficult to understand by a high proportion of participants, and the model fit was improved by removing it. Consequently, age-related factors may add to linguistic and cultural issues in accounting for the poor performance of items describing coping with uncertainty through deliberative processes. Future studies involving adolescents from different countries may be helpful to better clarify this aspect.

Item loadings on the group factors also align with previous research in adult samples. Our study showed that some IUS-R items failed to load significantly on the Prospective IU factor (i.e., #1, #2, #4, and #6), and the rest had small loadings (i.e., #3, #5). Similarly, all studies using a bifactor model to address the factor structure of the IUS scales in adulthood found that the Prospective IU domain was weak (Bottesi et al., 2019b; Hale et al., 2016; Huntley et al., 2020; Lauriola et al., 2016; Shihata et al., 2018; Yao et al., 2021). However, the only study using the IUS-R (Bottesi et al., 2019b) reported a slightly stronger Prospective IU factor than the other studies because all factor loadings were statistically significant, though only three items had values greater than 0.30. Therefore, although the IUS-R is intended to be used across the lifespan, our findings suggest that the Prospective IU factor is inconsistent in adolescents, even more so than in adults, at least in the Italian context. Further item refinement is perhaps needed to obtain a more reliable and theoretically sound Prospective IU factor in adults and young people.

The Inhibitory IU factor was robust, with only one item (i.e., #12) failing to yield a statistically significant loading. Nevertheless, in line with previous research, this factor only accounted for a small portion of variance, far less than the General factor (Bottesi et al., 2019b; Hale et al., 2016; Lauriola et al., 2016; Shihata et al., 2018). Recent studies have also shown that Inhibitory IU is unreliable in nonclinical (Huntley et al., 2020; Yao et al., 2021) and GAD (Wilson et al., 2020) adult samples. Therefore, the Inhibitory IU subscale should be used with caution when assessing IU in the adolescent population. Some of the abovementioned studies also detected item #12 as problematic (Bottesi et al., 2019b; Hale et al., 2016; Huntley et al., 2020; Lauriola et al., 2016; Shihata et al., 2018). This item is likely not fully represent the Inhibitory IU domain, neither in adults nor in adolescents. In fact, unlike the other items describing a “behavioral paralysis” in the face of uncertainty (Birrell et al., 2011), item #12 refers to an active attempt to escape from uncertain situations (“I must get away from all things I am unsure of”). Consequently, to strengthen the Inhibitory IU factor, item #12 should be revised by making its content more consistent with the scale’s overall meaning.

In keeping with the above-discussed findings, our study evidenced high ω reliability coefficients for all factors in the bifactor model. However, while we found a slight difference between the ω and ω_h_ coefficients for the General factor, the same difference was remarkable for the Inhibitory and Prospective IU factors. These results prove that the two group factors do not contribute considerably to the reliability of the corresponding subscales, which, on the contrary, derive most of their reliable variance from the General factor. This pattern and the common variance accounted for by the general factor recommend using a total score instead of subscale scores (Rodriguez et al., 2016), a conclusion also reached by several studies on the adult population (Hale et al., 2016; Huntley et al., 2020; Shihata et al., 2018). In sum, group factors are needed to model the IU construct to account for imperfect items or systematic variance in IU side contents; however, they have little relevance for psychological assessment.

An application of the IUS-R should be to assess how IU develops from childhood to adulthood. Establishing measurement invariance is crucial for disentangling whether inferences based on the IUS-R scores reflect variability in the IU construct or item responses unrelated to IU. Our study supported the bifactor model’s configural and metric invariance in different age and sex groups. These findings ensure the unbiased comparison of correlations involving the IUS-R and other criteria instruments in preadolescents, early adolescents, middle adolescents, and samples of girls and boys. Unfortunately, the scalar invariance was not fully supported by age and sex. The former result suggests that mean differences in the IUS-R scores are unbiased only from 14 years old and up. The latter means that girls and boys may differ not necessarily in IU, but also in how they respond to specific items. We tested age and sex differences in the IUS-R total score, showing that the preadolescent group was significantly more intolerant of uncertainty than the two adolescent groups. At the same time, girls obtained higher scores than boys. In light of the partial scalar invariance, we cannot be sure that the mean differences between age groups correspond to a higher level of IU in preadolescents. Still, it may also stem from how the youngest participants interpreted items #2, #5, and #12. Likewise, gender differences may arise from different meanings that girls and boys attached to items #1, #7, #8, and #12. While we have already discussed item #12 as problematic from several perspectives, the contents of the remaining items need to be re-evaluated for their use under age 14 or to study sex differences during adolescence.

Some authors have argued that mean differences can be meaningful under partial scalar invariance, if most items in a scale are invariant (Steenkamp & Baumgartner, 1998; Steinmetz, 2013). Our findings suggest that IU may be decreasing from preadolescence to adulthood if these authors are correct. Indeed, preadolescents were significantly more intolerant of uncertainty than adolescents, who, in turn, obtained higher scores on the IUS-R compared to the Italian adult normative sample (see Bottesi et al., 2019b). In general, younger participants may be high in IU because they frequently cope with many new, changeable, and uncontrollable events, scattered by uncertainty (Casey et al., 2010; Read et al., 2013); therefore, IU may be a phase-specific feature of adolescence itself. The relatively high scores we found in preadolescents and adolescents are not frequently observed in normative adult samples, wherein such high scores indicate a risk of developing psychopathological symptoms. However, considering that our data were collected in the school setting, thus with a low prevalence of psychopathology, high scores assume a different meaning. Adolescents may perceive uncertainty and difficulties managing it as more “normal” than adults, thus experiencing emotional, behavioral, and cognitive responses to uncertainty as less negative. Consequently, high IU scores at younger ages may not necessarily reflect an increased risk of developing a mental disorder, further supported by the absence of significant direct associations between IU and general psychological well-being that emerged from correlational analyses. However, future research in clinical samples is needed to shed light on the relationship between IU and the onset of mental disorders in teenagers.

Finally, IU scales are expected to yield moderate-strong correlations with internalizing symptoms (e.g., Boelen et al., 2010; Bottesi et al., 2019b; Comer et al., 2009; Cornacchio et al., 2018; Osmanağaoğlu et al., 2021; Wright et al., 2016). Accordingly, we found moderately high correlations between the IUS-R total score and internalizing dimensions, namely anxiety, depression, social problems, and withdrawal; therefore, our study provides additional support to the well-established association between IU and internalizing problems in adolescents. With specific regard to anxiety-related dimensions, regression analyses showed that the generalized anxiety scale of the SAFA-A accounted for the largest unique amount of IUS-R variance, thus suggesting that IU may be a core feature of GAD in adults and adolescents. Although significant in regression analyses, social and school anxiety scores accounted for a limited amount of the IUS-R variance. This finding suggests that IU may be implicated in symptoms pertaining to all anxiety-based disorders, but not in equal measure in all. The relationship between IU and social, school, and separation anxiety symptoms in teenagers may be moderated by other cognitive, emotional, or social variables. For example, previous studies found that several vulnerability factors (e.g., looming cognitive style, fear of negative evaluation, anxiety sensitivity) are involved in the relationship between IU and anxiety-related problems (including social anxiety) in adults (e.g., Boelen & Reijntjes, 2009; Riskind et al., 2000). Similarly, together with IU, other factors may have a bearing on the onset and maintenance of social, separation, and school anxiety in adolescence.

Lastly, our findings supported an adequate divergent validity of the IUS-R. Although we expected negative correlations between scales assessing general psychological well-being and the IUS-R, the fact that we ascertained significance only with the P Scale and still modest in magnitude is reasonable. Indeed, the PWB scale does not measure the experience of positive vs. negative mood states, but an existential form of well-being associated with realizing personal potential (i.e., eudaimonic well-being). Accordingly, a recent cluster analysis study showed that hedonic and eudaimonic well-being could be dissociated (Pancheva et al., 2021). While low hedonic well-being is undoubtedly a feature that might characterize adolescents’ anxious or depressive states (i.e., anhedonia), of which IU might be the foundation, the same cannot be said for low eudemonic well-being. Therefore, the absence of association with the IUS-R may suggest that adolescents might not experience negative emotions towards the low realization of personal potential as adults may do. Furthermore, a small negative correlation between the IUS-R and the P Scale emerged. Again, this is in line with our hypothesis since a positive attitude towards life is rather uncommon in people with high IU, who instead tend to find everyday life uncertain situations as upsetting and undesirable.

## Limitations and future directions

Some limitations characterize the present study. First, our results cannot be fully generalized to the Italian preadolescent and adolescent population despite the relatively large sample size. Indeed, participants were recruited exclusively in northern Italy; future studies may involve youth residing in central and southern regions to maximize the sample’s representativeness. Second, we cannot exclude that the length of the survey may have somewhat interfered with the reliability of the completion. For instance, boredom should be considered, given that adolescents are generally more prone to being bored (e.g., Caldwell et al., 1999). Then, we did not include a parent-report assessment of IU. We aimed to provide information about the factor structure and psychometric properties of the self-report version of the IUS-R. Still, we acknowledge that availing of a parent-report measure could have further informed our results. Last, our study lacks a clinical sample of adolescents and, consequently, present findings may not generalize to young people with full-blown psychopathologies. Future research examining the measurement invariance of the IUS-R factor model across nonclinical and clinical samples of adolescents is recommended.

Despite its limitations, our study supports using the IUS-R to measure IU across the lifespan. In addition, consistently with the extant literature, our findings pointed out several issues regarding the measurement of Prospective and Inhibitory IU. To note, previous research in adults and adolescents showed that the two IU components have distinct associations with different strategies used to manage uncertain situations, as well as with psychopathological constructs (e.g., Boelen et al., 2010; Bottesi et al., 2019a; Hong & Lee, 2015; Wright et al., 2016). Further revisions of both scales are thereby recommended to clarify the role of the IU components in influencing the performance of particular coping strategies to deal with uncertainty and the development of specific psychological problems. Specifically, aligned with recent recommendations, any future refinement of the IUS-R may be grounded on a theory-driven approach to adequately capture any group factors potentially underlying the general construct (Bottesi et al., 2019b).

Several clinical implications can be traced from the current results. A noteworthy aspect brought to light by the present study regards the transdiagnostic nature of IU, which was found to be significantly associated with thought-related issues and externalizing symptomatology (i.e., aggressive behavior and attention problems). In particular, the thought-related problems scale of the YSR encompasses a wide range of symptoms, such as self-harming behaviors, obsessive-compulsive symptoms, psychotic symptoms, and sleep problems. Overall, these results are consistent with the literature; in fact, although IU is notoriously a factor spanning internalizing symptoms (Carleton, 2016a, b), recent studies have suggested that IU may contribute to externalizing psychopathology as well (e.g., Bottesi et al., 2021). Therefore, our findings may indicate that IU may not be only associated with anxiety and other internalizing dimensions, but also with a broad spectrum of psychopathological symptoms and conditions. It is possible that, under certain individual and environmental conditions, IU poses a risk for the development of psychological disorders in teenagers. This issue is vital for future research, which should involve a clinical population to pinpoint any differences vis-à-vis the general adolescent population and clarify the link between IU, psychological well-being, and mental disorders. Moreover, potential protective factors should also be considered to study how they influence the path from IU to the onset of psychological disorders and include them in the implementation of preventive programs aimed at the adolescent population.

In conclusion, because the multidimensionality of the IUS-R does not appear to be substantial in adolescents, the present study recommends researchers to model the factorial structure of the IUS-R according to a bifactor approach and urges clinicians to use the total score instead of subscale scores when assessing adolescents. Since IU can be a defining characteristic of adolescents and simultaneously a transdiagnostic vulnerability factor, it seems crucial to rely on the IUS-R to determine its clinical levels and further investigate IU’s relationships with psychopathological constructs in this age group. In particular, IU is a well-known risk factor for the onset of anxiety disorders, which are the psychological problems with the highest prevalence rate among teenagers (e.g., De Girolamo et al., 2012). Consequently, it would be beneficial to widen knowledge on the relationship between IU and anxiety-based disorders and include the IUS-R as an outcome measure in treatment programs for adolescent anxiety. Lastly, taking a preventive approach, a thorough study of IU in teenagers would enable the design and development of early interventions to prevent maladaptive outcomes in such a vulnerable population; hence the importance of the IUS-R as a tool capable of reliably measuring IU in adolescents and a range of settings.

## Electronic supplementary material

Below is the link to the electronic supplementary material.

Supplementary Material 1

## Data Availability

The data that support the findings of this study are available from the corresponding author, G.B., upon reasonable request.
