# Supplementary material for: The assessment of Intolerance of uncertainty in youth: An examination of the Intolerance of Uncertainty Scale-Revised in Italian nonclinical boys and girls
Source: Res Child Adolesc Psychopathol. 2022 Jul 2;51(2):209–22. doi: 10.1007/s10802-022-00944-y (PMC9867688; doi:10.1007/s10802-022-00944-y)
Supplement: Supplementary file 1 — Supplementary Material 1 [file 10802_2022_944_MOESM1_ESM.docx]

**The assessment of Intolerance of uncertainty in youth: An examination of the Intolerance of Uncertainty Scale-Revised in Italian nonclinical boys and girls**

**Supplementary Information**

**Table S1**

*Mean and Standard Deviation (SD) of the Scores on the Administered Tools (Except for the IUS-R)*

| Scale | *N* | *M* | *SD* |
| --- | --- | --- | --- |
| PWB Total | 844 | 72.4 | 15. 4 |
| P Scale | 858 | 28 | 5.52 |
| YSR Anxiety/Depression | 860 | 7.33 | 4.81 |
| YSR Withdrawal/Depression | 860 | 4.19 | 3.33 |
| YSR Somatic Complaints | 854 | 3.78 | 3.38 |
| YSR Social Problems | 859 | 4.45 | 3.28 |
| YSR Thought-related Problems | 859 | 5.82 | 4.11 |
| YSR Attention Problems | 861 | 6.43 | 3.43 |
| YSR Rule-Breaking Behavior | 859 | 4.58 | 4.07 |
| YSR Aggressive Behavior | 859 | 8.94 | 5.21 |
| YSR Externalizing Problems | 859 | 13.5 | 8.45 |
| YSR Internalizing Problems | 859 | 15.3 | 9.70 |
| SAFA-A Generalized Anxiety | 836 | 9.83 | 5.70 |
| SAFA-A Tension/Uneasiness | 850 | 4.93 | 3.33 |
| SAFA-A Preoccupation/Apprehension | 844 | 5.61 | 3.14 |
| SAFA-A Social Anxiety | 844 | 7.58 | 4.51 |
| SAFA-A Separation Anxiety | 823 | 7.48 | 4.32 |
| SAFA-A School Anxiety | 841 | 7.57 | 4.90 |

IUS-R = Intolerance of Uncertainty Scale – Revised; PWB = Psychological Well-Being; P Scale = Positivity Scale; YSR= Youth Self Report 11-18; SAFA-A= Self-Administered Psychiatric Scales for Children and Adolescents – Anxiety Scale.

**Table S2**

*Pearson's r Correlations Between the IUS-R and the Other Questionnaires*

| Scale | IUS-R | | |
| --- | --- | --- | --- |
| PWB | | -.01 |  |
| P Scale | | -.17 | *** |
| YSR Externalizing Problems | | .10 | ** |
| YSR Internalizing Problems | | .47 | *** |
| YSR Anxiety/Depression | | .51 | *** |
| YSR Withdrawal/Depression | | .36 | *** |
| YSR Somatic Complaints | | .28 | *** |
| YSR Social Problems | | .41 | *** |
| YSR Thought-related Problems | | .30 | *** |
| YSR Attention Problems | | .26 | *** |
| YSR Rule-Breaking Behaviors | | -.01 |  |
| YSR Aggressive Behaviors | | .17 | *** |
| SAFA-A Tension/Uneasiness | | .46 | *** |
| SAFA-A Preoccupation/Apprehension | | .53 | *** |
| SAFA-A Generalized Anxiety | | .54 | *** |
| SAFA-A Separation Anxiety | | .31 | *** |
| SAFA-A Social Anxiety | | .38 | *** |
| SAFA-A School Anxiety | | .44 | *** |
| *Note.* ** *p* < .01, *** *p* < .001  IUS-R = Intolerance of Uncertainty Scale-Revised; PWB = Psychological Well-Being; P Scale = Positivity Scale; YSR= Youth Self Report 11-18; SAFA-A= Self-Administered Psychiatric Scales for Children and Adolescents – Anxiety Scale | | | |

**Table S3**

*Summary of regression analyses. Panel a: General psychological well-being scales (PWB, P Scale), Panel b: Internalizing symptoms scales (SAFA-A Tension/Uneasiness, Preoccupation/Apprehension, Generalized Anxiety, Separation Anxiety, Social Anxiety, School Anxiety), and Panel c: Clinical Syndrome Scales (YSR Anxiety/Depression, Withdrawal/Depression, Somatic Complaints, Social Problems, Thought-related Problems, Attention Problems, Rule-Breaking Behaviors, Aggressive Behaviors) predict the IUS-R score.*

**Panel a)**

| **Predictor** | | **Estimate** | | **SE** | | **t** | | **p** | | **Stand. Estimate** |  |
| --- | --- | --- | --- | --- | --- | --- | --- | --- | --- | --- | --- |
| Intercept | | 40.53 | | 3.46 | | 11.73 | | < .001 | |  |  |
| PWB Total | | -.02 | | .05 | | -.43 | | .668 | | -.01 |  |
| P Scale | | -.27 | | .06 | | -4.68 | | < .001 | | -.16 |  |
| **R** | **R²** | | **F** | | **df1** | | **df2** | | **p** | | |
| 0.16 | 0.03 | | 11.02 | | 2 | | 837 | | < .001 | | |

*Note.* PWB = Psychological Well-Being; P Scale = Positivity Scale

**Panel b)**

| **Predictor** | | | | **Estimate** | **SE** | | **t** | **p** | | **Stand. Estimate** | |
| --- | --- | --- | --- | --- | --- | --- | --- | --- | --- | --- | --- |
| Intercept | | | | 21.04 | .66 | | 32.01 | < .001 | |  | |
| SAFA-A Tension/Uneasiness | | | | -.71 | .47 | | -1.50 | .135 | | -.26 | |
| SAFA-A Preoccupation/Apprehension | | | | -.13 | .52 | | -.26 | .796 | | -.05 | |
| SAFA-A Generalized Anxiety | | | | 1.11 | .52 | | 2.15 | .032 | | .68 | |
| SAFA-A Social Anxiety | | | | .27 | .07 | | 3.96 | < .001 | | .13 | |
| SAFA-A Separation Anxiety | | | | .08 | .07 | | 1.08 | .281 | | .04 | |
| SAFA-A School Anxiety | | | | .19 | .08 | | 2.37 | .018 | | .10 | |
| **R** | **R²** | **F** | **df1** | | | **df2** | | | **p** | |  |
| .58 | .34 | 68.16 | 6 | | | 805 | | | < .001 | |  |

*Note.* SAFA-A= Self-Administered Psychiatric Scales for Children and Adolescents – Anxiety Scale

**Panel c)**

| **Predictor** | | | | **Estimate** | **SE** | | **t** | **p** | | **Stand. Estimate** | |
| --- | --- | --- | --- | --- | --- | --- | --- | --- | --- | --- | --- |
| Intercept | | | | 24.26 | 0.65 | | 37.51 | < .001 | |  | |
| YSR Anxiety/Depression | | | | .72 | .09 | | 8.13 | < .001 | | .37 | |
| YSR Withdrawal/Depression | | | | .07 | .12 | | .56 | .573 | | .02 | |
| YSR Somatic Complaints | | | | -.01 | .10 | | -.11 | .916 | | .00 | |
| YSR Social Problems | | | | .30 | .13 | | 2.37 | .018 | | .11 | |
| YSR Thought-related Problems | | | | .15 | .10 | | 1.52 | .130 | | .07 | |
| YSR Attention Problems | | | | .25 | .10 | | 2.45 | .015 | | .09 | |
| YSR Rule-Breaking Behavior | | | | -.33 | .09 | | -3.64 | < .001 | | -.15 | |
| YSR Aggressive Behavior | | | | -.03 | .08 | | -.40 | .692 | | -.02 | |
| **R** | **R²** | **F** | **df1** | | | **df2** | | | **p** | |  |
| .53 | .28 | 40.9 | 8 | | | 840 | | | < .001 | |  |

*Note.* YSR = Youth Self Report 11-18
